# A signaling complex of adenylate cyclase CyaC of *Sinorhizobium meliloti* with cAMP and the transcriptional regulators Clr and CycR

**DOI:** 10.1186/s12866-023-02989-5

**Published:** 2023-08-26

**Authors:** Robin Klein, Jannis Brehm, Juliane Wissig, Ralf Heermann, Gottfried Unden

**Affiliations:** Institute of Molecular Physiology (imP), Microbiology and Biotechnology, Johannes Gutenberg University, Biocenter II, Hanns-Dieter-Hüsch-Weg 17, 55128 Mainz, Germany

**Keywords:** Second messenger, CyaC, Cyclic AMP, TetR-type regulator, cyclic-AMP-receptor protein CRP, *Sinorhizobium meliloti*, *Ensifer meliloti*

## Abstract

**Background:**

Adenylate cyclases (ACs) generate the second messenger cyclic AMP (cAMP), which is found in all domains of life and is involved in the regulation of various cell physiological and metabolic processes. In the plant symbiotic bacterium *Sinorhizobium meliloti*, synthesis of cAMP by the membrane-bound AC CyaC responds to the redox state of the respiratory chain and the respiratory quinones. However, nothing is known about the signaling cascade that is initiated by cAMP produced by CyaC.

**Results:**

Here, the CRP-like transcriptional regulator Clr and the TetR-like regulator CycR (TR01819 protein) were identified to interact with CyaC using the bacterial two-hybrid system (BACTH), co-sedimentation assays, and surface plasmon resonance spectroscopy. Interaction of CycR with Clr, and of CyaC with Clr requires the presence of cAMP and of ATP, respectively, whereas that of CyaC with CycR was independent of the nucleotides.

**Conclusion:**

The data implicate a ternary CyaC×CycR×cAMP-Clr complex, functioning as a specific signaling cascade which is formed after activation of CyaC and synthesis of cAMP. cAMP-Clr is thought to work in complex with CycR to regulate a subset of genes of the cAMP-Clr regulon in *S. meliloti*.

**Supplementary Information:**

The online version contains supplementary material available at 10.1186/s12866-023-02989-5.

## Background

Adenylate cyclases (ACs) generate the second messenger cyclic AMP (cAMP), which plays an important role in signaling in all domains of live [1, 2]. The ACs have a broad significance for the regulation of diverse processes in cell physiology and metabolism. CyaC (SMc01818 protein) of *Sinorhizobium (Ensifer) meliloti* belongs to the bacterial class III ACs, which are homodimers, are mostly membrane-bound and have a large variation in domain composition [3–5]. CyaC comprises a hexa-helical transmembrane domain (6TM) binding two heme-B molecules. likely in a similar arrangement as bacterial di-heme-B succinate dehydrogenases and fumarate reductases [5–7]. The heme-B molecules render the activity of CyaC redox sensitive [5]: heme-B of CyaC can be oxidized or reduced by ubiquinone, which affects the cyclase activity of CyaC.

In contrast to the mode of sensing by CyaC, nothing is known about the downstream signaling cascade triggered by CyaC. In particular, *S. meliloti* contains 28 ACs [8, 9], which are supposed to feed cAMP into individual signaling chains. It is not clear how the signaling chains are organized in order to allow specific signaling within different signaling chains since they all use cAMP as the mediator. Interestingly, *S. meliloti* contains a transcriptional activator designated as Clr (Smc02175), which belongs to the family of CRP-like transcriptional regulators with cAMP-stimulated DNA-binding activity [10]. In *S. meliloti*, approximately 150 genes are under transcriptional control of cAMP-Clr [9, 10]. Clr is part of a signaling cascade starting from cyclases CyaD1, CyaD2 and CyaK that control the symbiotic interaction of *S. meliloti* with the plant host *Medicago sativa* [10, 11]. Perception of a plant-derived signaling molecule by CyaK requires the predicted β-barrel outer membrane protein NsrA [12]. Understanding the molecular mechanisms and the interrelation of the CyaD1, CyaD2, and CyaK-cAMP-Clr signaling cascades is still limited. However, there is first indication that Clr function is not restricted to an interaction with CyaD1, CyaD2 and CyaK, but also has a further role in the transcriptional regulation of other genes [9, 10, 13].

In bacteria, genes for functionally or structurally linked proteins of metabolic or signaling pathways are often clustered [14]. Interestingly, *cyaC* (*smc01818*) is preceded by a gene (*smc01819*) that encodes a putative transcriptional regulator (TR01819) of the TetR family with an *N*-terminal Helix-Turn-Helix DNA binding motif. TetR regulators often bind small-molecular ligands as signals for modulating DNA-binding and regulation of gene expression [15]. However, the target genes of TR01819 are unknown.

To get insight into the components and function of the CyaC-related signaling pathway in *S. meliloti*, we investigated whether Clr or TR01819 interact with CyaC, and whether this putative interaction is cAMP-dependent. Defining interaction partners of CyaC will help to identify the components of the cAMP-dependent signaling route starting from CyaC, and to understand its relation to other cAMP-dependent signaling cascades. Since TR01819 showed close and specific interaction with CyaC and with Clr, the protein was renamed as CycR to indicate the supposed role in CyaC-dependent signaling.

## Materials and methods

### Bacterial strains and growth conditions

The *E. coli* K12 strains and plasmids are listed in Table 1. The molecular genetic methods, including cloning, generation of *lacZ* fusions, DNA isolation, and manipulations were performed according to standard procedures [16, 17]. Bacteria were grown aerobically or anaerobically at 37 °C or 30 °C in minimal medium M9 [17], enriched M9 (eM9) [18] or LB broth [17] with the respective carbon source indicated for the individual experiments. Growth was measured as optical density at 578 nm (OD_578_). For the BACTH assays [19], strain *E. coli* BTH101 was transformed with the respective plasmids encoding the T18 and T25 fragments of AC_Bp_. Derivatives of pUT18 and pUT18C encoding the domain T18 for N-or C-terminal fusion, and pKT25 and pKNT25 encoding the T25 fragment for C- and N-terminal fusion, respectively, were used for producing the fusion proteins. The pUT18- and pKT25-derived plasmids encode Ap^R^ and Km^R^, respectively, and the corresponding antibiotics were used during cultivation to maintain the plasmids. For the BATCH assays, the bacteria were grown anaerobically in LB broth with 20 mM DMSO which produces highest activities [5], in microtiter plates to an OD_578_ 0.6 to 0.9. The β-galactosidase assays were performed as described elsewhere [20, 21]. The β-galactosidase activities are presented as the mean (with standard deviation) from at least two biological and four technical replicates each.

**Table 1:** Strains of *E. coli* and plasmids

Strain	Genotyp	Reference
BL21 (DE3)	F– *ompT gal dcm lon hsdS*_*B*_(*r*_*B*_^*–*^*m*_*B*_^*–*^) *λ*(DE3 [*lacI lacUV5-T7p07 ind1 sam7 nin5*]) [*malB*^*+*^]_*K−12*_*(λ*^S^) pLysS[*T7p20 ori*_*p15A*_](Cam^R^)	[38]
BTH101	F- *cya-99*, *araD139*, *galE15*, *galK16*, *rpsL1, hsdR2*, *mcrA1*, *mcrB1* (Str^R^)	[19]
C43 (DE3)	Derivative of BL21 (DE3), F − *ompT, gal, dam, lon, hsdS*_*B*_(*r*_*B*_^*-*^*m*_*B*_^*-*^) *λ*(DE3 [*lacI lacUV5-*T7 *gene 1 ind1 sam7 nin5*])	[39]
**Plasmid**	**Genotype**	**Reference**
pMW3043	pET28a, but with His_6_-CyaC	This work
pMW3065	pASK-IBA3, but with CycR-Strep	This work
pMW3084	pET28a, but with His_6_-Clr	This work
pMW3085	pASK-IBA3plus, but with Clr-Strep	This work
pKT25	pSU40, but for C-terminal T25 fusion, Km^R^	[19]
pKNT25	pSU40, but for N-terminal T25 fusion, Km^R^	[19]
pUT18	pUC19, but for N-terminal T18 fusion, Ap^R^	[19]
pUT18C	pUC19, but for C-terminal T18 fusion, Ap^R^	[19]
His_6_-pKT25	pKT25 derivative, but with His_6_-Tag	[40]
His6-pKNT25	pKNT derivative, but with His_6_-Tag	[20]
His6-pUT18	pUT18 derivative, but wih His_6_-Tag	[20]
His_6_-pUT18C	pUT18C deriative, but with His_6_-Tag	[40]
pKNT25-zip	pKNT25, but T25-zip, Km^R^	[19]
pUT18-zip	pUT18, but T18-zip, Ap^R^	[19]
pMW689	His_6_-pUT18 derivative with CyaC^#^ [CyaC(K410/T484A)]	This work
pMW692	His_6_-pKNT25 derivative with CyaC^#^ [CyaC(K410/T484A)]	This work
pMW690	His_6_-pUT18 derivative with CyaC^##^ [CyaC(R495A/N491A)]	This work
pMW691	His_6_-pKNT25 derivative with CyaC^##^ [CyaC(R495A/N491A)]	This work
pMW1424	pUT18C, butT18-NreC (*S. carnosus*), Ap^R^	[41]
pMW1439	pUT18, but NreC-T18 (*S. carnosus*), Ap^R^	[41]
pMW1777	pKNT25, but NreC-T25 (*S. carnosus*), Km^R^	[41]
pMW1780	pKT25, but T25-NreC (*S. carnosus*), Km^R^	[41]
pMW2958	His_6_-pKNT25, but CycR-T25, Km^R^	This work
pMW2959	His_6_-pKT25, but T25-CycR, Km^R^	This work
pMW2961	His_6_-pUT18C, but T18-CycR Amp^R^	This work
pMW3087	pUT18, but Clr-T18, Amp^R^	This work
pMW3088	pUT18C, but T18-Clr, Amp^R^	This work
pMW3090	pKNT25, but Clr-T25, Km^R^	This work

### Protein production and purification

*E. coli* C43(DE3) with plasmid pMW3043 and *E. coli* BL21 (DE3) with pMW3084 were used for overproduction of His_6_-CyaC and Clr-His_6_. The bacteria were grown in 2 to 4 × 400 ml LB broth supplemented with 50 mg/l kanamycin in baffled 2 L flasks at 37 °C with shaking (180 rpm) and incubated until the cultures reached an OD_578_ of 0.8 and 0.5, respectively. After induction of gene expression using 0.5 mM isopropyl-β-D-thiogalactopyranoside (IPTG), the bacteria were incubated for further 4 h at 30 °C. Then, the cultures were harvested by centrifugation (4 °C, 20 min, 6,000 rpm) and washed with buffer. The wet cells were frozen in liquid nitrogen and stored at -80 °C, or used directly for protein purification. For the purification of His_6_-CyaC, 5 to 10 g wet cells were resuspended in 20 ml buffer 1 (50 mM K-phosphate, 150 mM NaCl, pH 7.5) in 50 ml tubes. The suspension was passed three times through a French pressure cell and centrifuged first at 8.000 rpm to remove cell debris and then at 50,000 rpm to separate the cytosolic from the membrane fraction. After washing, His_6_-CyaC was extracted from the membranes fraction using a buffer containing 1% n-dodecyl-β-D-maltoside as a detergent. His_6_-CyaC was purified by Ni^2+^-NTA chromatography as described earlier [5]. Finally, His_6_-CyaC was eluted from the column in buffer [50 mM K-phosphate, 150 mM NaCl, 0.05% (w/v) n-dodecyl-β-D-maltoside, 250 mM L-histidine]. For the overproduction of Clr-His_6_, the French press homogenate was centrifuged at 18,000 rpm for 30 min at 4 °C. The supernatant was directly subjected ti Ni^2+^-NTA chromatography. The protein was finally eluted using buffer containing 50 mM Na-phosphate, 300 mM NaCl and 250 mM imidazole at pH 8.0.

To produce Clr-Strep and CycR, *E. coli* BL21(DE3) carrying plasmid pMW3085 or pMW3065, respectively, was grown in 6 × 400 ml LB broth with ampicillin (100 mg/L) to an OD_578_ of 0.5. Then, 200 ng/ml anhydrotetracycline was added to induce synthesis of Clr-Strep or CycR, and the culture was aerobically incubated for 5 h at 30 °C. The cells were harvested as described for the preparation above and resuspended in buffer W (100 mM Tris/HCl, 150 mM NaCl, 1 mM EDTA at pH 8.0). Clr-Strep was purified from a French press cell homogenate via chromatography using Strep-Tactin resin (IBA Life Sciences, Göttingen), and eluted from the column with buffer W containing 2.5 M D-desthiobiotine.

### Protein co-purification

The in vitro interaction between CyaC, Clr and CycR was tested via co-elution of respective protein pairs using HisMagnetic beads (Promega) as described before [22]. In each experimental set-up one of the proteins was provided with a His_6_-tag. HisMagnetic beads (5 µl) were equilibrated with 200 µl buffer 1 (50 mM K-phosphate, 150 mM NaCl, in the presence or absence of 0.5 mM cAMP and 3.5 mM ATP as indicated, at pH 7.5). The His_6_-and the Strep-tagged proteins (2 µM each in 500 µl buffer 1) were incubated at 30 °C and 1,000 rpm for 10 min, mixed with equilibrated HisMagnetic beads (5 µl beads in 200 ml buffer 1) and incubated for further 5 min at 30 °C under gentle movement (1,000 rpm). The beads were washed twice with 300 µl buffer 1, and the proteins were eluted with 20 µl buffer 1 containing imidazole (0.5 M) for 5 min. Eluted samples were mixed with SDS-PAGE sample buffer and analyzed via SDS-PAGE using 15% gels and finally detected by Western blotting using the respective antibodies [21, 23]. Immunostaining was performed with horse-radish peroxidase (HRP)-coupled anti-His-HRP, anti-Strep-HRP, and anti-IgG-mouse-HRP polyclonal antiserum (Sigma-Aldrich) or with anti-PhoA antibodies (produced in mouse, Sigma-Aldrich). For visualization the blots were subjected to a chemiluminescent substrate (HRP; Merck Millipore) and exposed on X-ray films (Advansta).

### Surface plasmon resonance spectroscopy (SPR)

SPR assays were performed in a Biacore T200 device using Series S CM5 carboxymethyl dextran sensor chips (Cytiva, Freiburg) that had been immobilized with StrepTactin® resin (IBA, Göttingen) following the instructions manual of the distributer using N-ethyl-N-(3-dimethylaminopropyl)carbodiimide hydrochloride and N-hydroxysuccinimide using the standard amine-coupling protocol (Amine coupling kit, Cytiva). The experiments were performed in HBS-EP+ buffer [10 mM HEPES pH 7.4, 150 mM NaCl, 3 mM EDTA, 0.05% (v/v) detergent P20]. Free binding sites on the flow cells were saturated by injection of 1 M ethanolamine/HCl (pH 8.0). Preparation of chip surfaces was carried out at a flow rate of 10 µl/min. As first step, 200–220 RU Strep-ClrR were captured onto the second flow cell of the chip at a constant flow rate of 10 µl/min. The first flow cell was left uncaptured as control. Then, different concentrations (10 nM, 25 nM, 50 nM, 100 nM, 2 × 250 nM, 500 nM, 1.000 nM, 2.500 nM, and 5.000 nM, respectively) of His_6_-CyaC (in the presence or absence of 500 µM cAMP) were then injected at flow rate of 30 µl/min over both flow cells using a contact time of 360 s each following a 420 s dissociation time. All experiments were performed at 25 °C. Sensorgrams were recorded using Biacore T200 Control software 3.2 and analyzed with Biacore T200 Evaluation software 3.2 (Cytiva). The surface of flow cell 1 was used to obtain blank sensorgrams for subtraction of the bulk refractive index background. The referenced sensorgrams were then normalized to a baseline of 0. Spikes at the start and the end of the injections emerged from the run-time difference between the flow cells. Three biological replicates were performed.

## Results

### Interaction of CyaC, Clr and CycR in vivo

In order to identify potential candidates of a signaling cascade emanating from CyaC, the respective proteins were tested for physical interaction with CyaC, i.e. the cAMP-stimulated transcriptional activator Clr (Smc02175), and the TetR-like transcriptional regulator CycR (Smc protein 01819) that is encoded by gene *smc01819* preceding *cyaC* (*smc01818* gene).

**Fig. 1:**
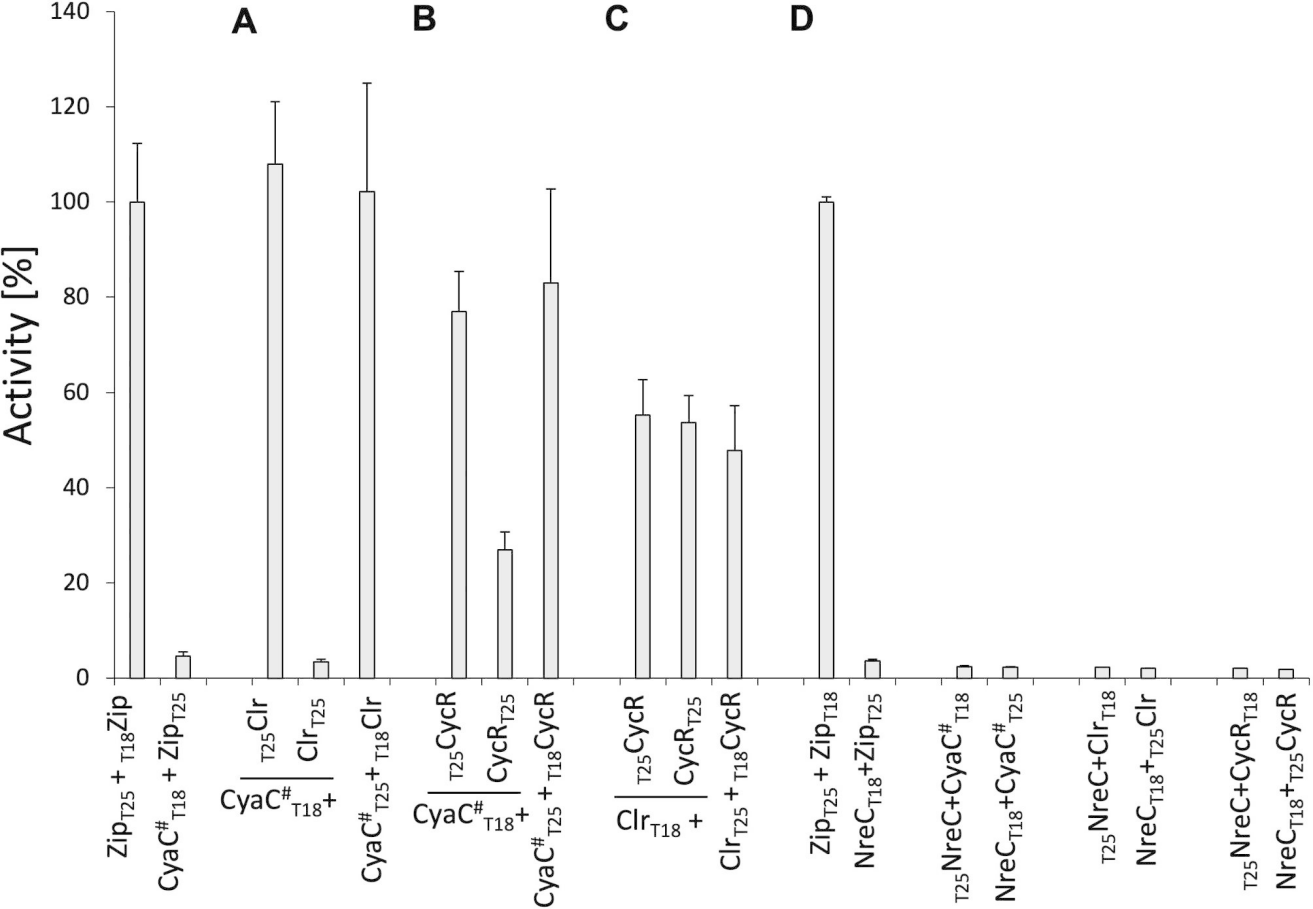
Interaction of CyaC, Clr and CycR tested by the BACTH system for the pairs CyaC^#^/ Clr **(A)**, CyaC^#^/CycR **(B)**, Clr / CycR **(C)**, and the non-related gene regulator NreC **(D)** in *E. coli *BTH101 Proteins CyaC^#^ (Cyac(K410A T484A), Clr and CycR were fused C- or N-terminally to the T18 or T25 fragments as indicated, and produced by pairwise co-expression of the corresponding genes from plasmids (Table 1). The Leu zipper (Zip) pair fused to T18 and T25 fragments, respectively, was used as the positive control, the pair Zip-T25 with CyaC^#^-T18 as the negative control. 100% activity corresponds to 1,450 Miller-Units in **(A)** and **(C)**, 2,700 Miller-Units in **(B)**, and 1.630 Miller-Units in **(D)**. Bacteria were grown anaerobically in LB medium supplemented with 20 mM dimethylsulfoxide. β-Galactosidase activities are given in Miller-Units MU [17] as the mean (with standard deviation) from at least two biological and four technical replicates each

First, the interaction of CyaC with Clr and CycR and was tested in vivo (Fig. 1AB) using the bacterial adenylate cyclase based two-hybrid system (BACTH) of *E. coli*. Fusions of CyaC and Clr with the T25 or T18 domains of *Bordetella pertussis* adenylate cyclase (AC_Bp_) were produced in *E. coli* BTH101 by cloning the *cyaC*, and *clr* genes, respectively, up- or down-stream the gene fragments for T18 and T25 in various combinations. For *cyaC*, a variant had to be used that produces an enzymatically inactive CyaC protein. Inactivation is required to allow measurement of the reporter cyclase activity of AC_Bp_ in the BACTH system without interference from the inherent adenylate cyclase activity of CyaC. For inactivation, amino acid residues Lys410 and Thr484 in the catalytic ATP site of the homodimer of CyaC [5, 24, 25] were replaced to obtain variant CyaC(K410A T484A), or CyaC^#^. Strains producing CyaC^#^ showed very low background AC (and consequently β-galactosidase) activity (Fig. 1A), which allows use of the variant for testing the restoration of *B. pertussis* AC_Bp_ adenylate cyclase activity in the reporter strain. AC_Bp_ should be restored from the T18- and T25 domains of AC_Bp_ when the linked test proteins trigger interaction of the domains. Strains co-producing T18 and T25 fused to CyaC^#^ and to Clr (Fig. 1A), respectively, showed high restoration of AC_Bp_. The levels are as high as 108% of the positive control that is represented by fusions of T18 and T25 to a zipper protein. Figure 1A gives a selection of fusions most of which showed high interaction.

When the interaction of CyaC^#^ with CycR was analyzed by the same method, the fusions showed also a high degree of AC_Bp_ restoration (Fig. 1B), indicating that CycR interacts with CyaC, similar like Clr. To underpin the unexpected interaction of CyaC with both transcriptional regulators, a further set of fusions was used where the intrinsic AC activity of CyaC was inactivated by an alternative mutation, that is by replacing amino acid residues Arg495 and Asn491. The pair of residues stabilizes in class III AC enzymes, such as CyaC, the transition state of the dimer in catalysis [5, 24, 25]. As expected, derivative CyaC(R495A N491A), or CyaC^##^, was silent in the BACTH test strain, similar to the CyaC^#^ producing strain used in Fig. 1A and B, probably due to the loss of the intrinsic AC activity of the protein, but revealed activity of AC_Bp_ restoration when the pairs CyaC^##^×Clr and CyaC^##^×CycR were tested (data for CyaC^##^ not shown).

Altogether, the data indicate that Clr and CycR interact with CyaC when CyaC was applied in versions CyaC^#^ or CyaC^##^ which are compatible with the BACTH system. Consequently, interaction of Clr with CycR was tested in the BACTH system by providing Clr and CycR in suitable combinations of T18 and T25 fusions (Fig. 1C). Clr interacted with CycR in all fusions given by high activities in the BACTH assay, suggesting that CyaC, Clr and CycR interact with each other in vivo when the proteins are produced in *E. coli*. The interactions appear to be specific for the three regulators since neither CyaC, Clr or CycR showed any interaction with unrelated transcriptional regulators such as NreC (Fig. 1D).

### Interaction of Clr, CyaC and CycR in vitro: Effect of ATP, cAMP and activity state of CyaC

**Fig. 2:**
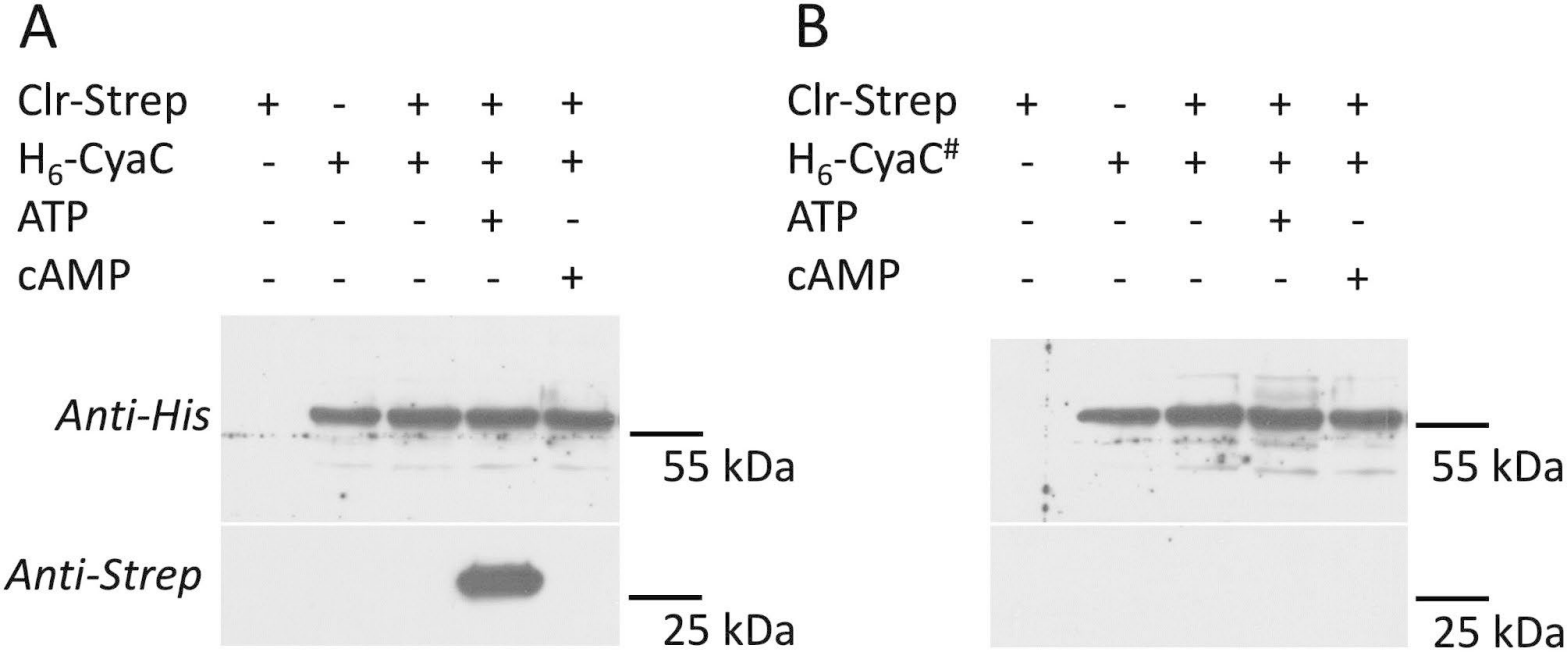
Co-sedimentation of Clr-Strep with **(A)** His_6_-CyaC and **(B)** His_6_-CyaC^#^ bound to magnetic beads. His_6_-CyaC (64 µg) or His_6_-CyaC^#^ (64 µg) were incubated in 500 µl binding buffer containing Clr-Strep (29.6 µg), ATP (3.5 mM) and cAMP (0.5 mM) as indicated. After separation of the beads and washing, the proteins associated with the beads were separated by SDS-PAGE and tested by Western-blotting for CyaC (anti-His_6_ antisera, upper panels) and Clr (anti-Strep antisera, lower panels) separately. All experiments were performed in two or more repeats. The positions of His_6_-CyaC (64.0 kDa) and Clr-Strep (29.6 kDa) in the Westernblot are indicated by the protein markers of 25 und 55 kDa. The full length blots are shown in Figure S1

The interaction of the proteins Clr, CyaC and CycR was characterized in vitro in more detail. First, the membrane-integral His_6_-CyaC was overproduced, solubilized in detergent, purified [5] and bound to magnetic beads carrying Ni^2+^-NTA. Binding of His_6_-CyaC to the magnetic beads was stable and allowed sedimentation of CyaC with the beads (Fig. 2A). When the beads with attached His_6_-CyaC were incubated with purified Clr protein as well, Clr did not sediment with the beads even when cAMP was present in the incubation mixture. However, presence of ATP stimulated co-sedimentation. When the same experiment was performed with the His_6_-CyaC^#^ variant, Clr no longer co-sedimented, neither in the absence or the presence of ATP (Fig. 2B). Therefore, binding of Clr to CyaC is specific and requires binding of ATP, or reaction of CyaC with ATP (i.e. cAMP formation). In the BACTH assay CyaC^#^ showed positive response to Clr (Fig. 1A), but the BACTH assay is not quantitative with respect to interaction strength and more permissive than the more discriminating co-sedimentation.

**Fig. 3:**
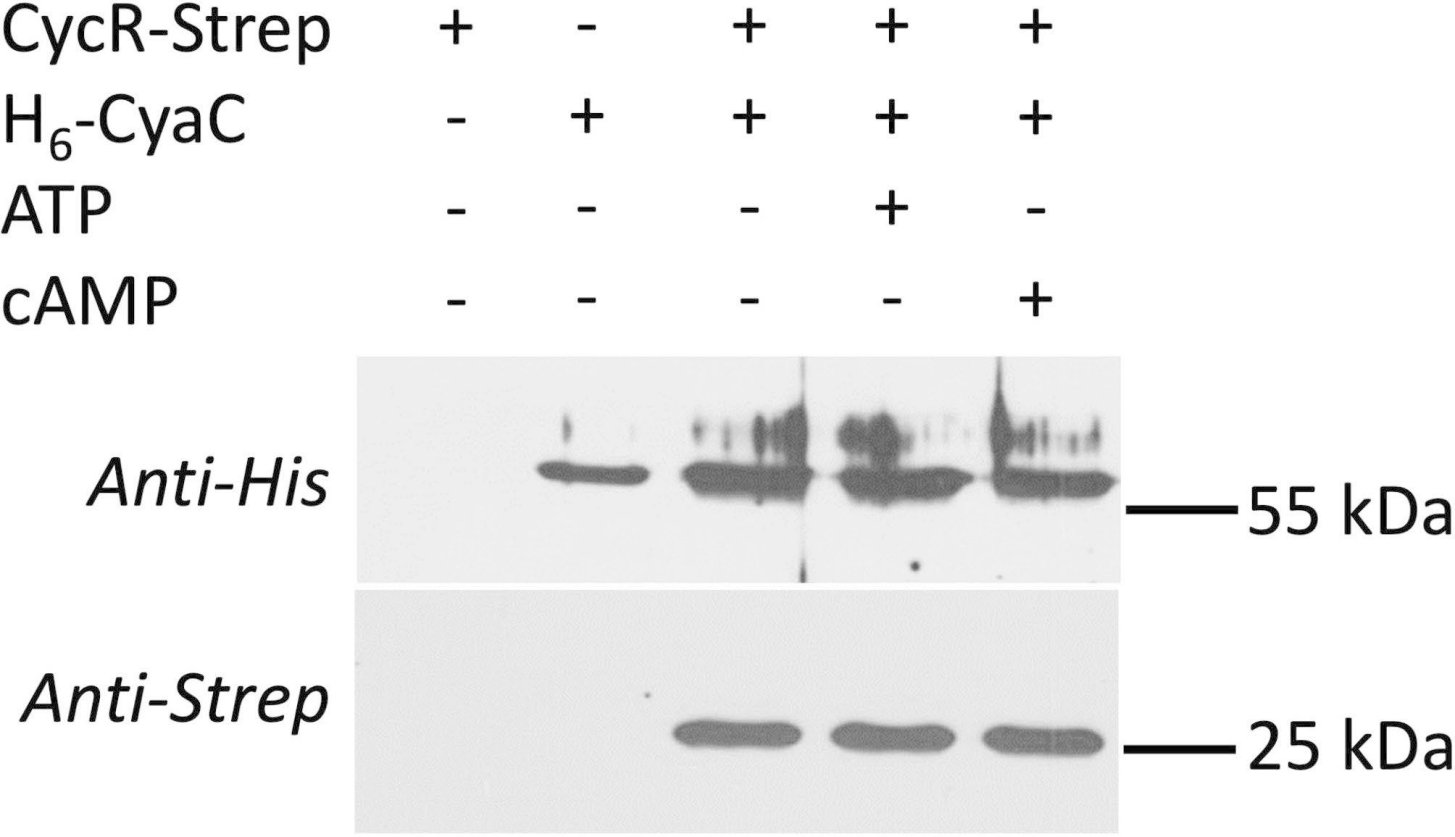
Co-sedimentation of CycR-Strep with His_6_-CyaC bound to magnetic particles. His_6_-CyaC (64 µg) was incubated in 500 µl binding buffer containing CycR-Strep (28.3 µg), ATP (3.5 mM) and cAMP (0.5 mM) as indicated. After separation of the beads and washing, the proteins associated with the beads were separated by SDS-PAGE and tested by Western-blotting for CyaC (anti-His_6_ antiserum, upper panel) and CycR (anti-Strep antiserum, lower panel) separately. Experiments were performed in two or more repeats. The positions of His_6_-CyaC (64.0 kDa) and CycR-Strep (28.3 kDa) in the Westernblot are indicated by protein markers of 25 und 55 kDa. The full length blots are shown in Figure S2

Interaction of CyaC with the transcriptional regulator CycR was tested by co-sedimentation after binding His_6_-CyaC to magnetic beads (Fig. 3). CycR co-sedimented whenever CyaC was bound to the beads, and neither ATP nor cAMP were required. These data reveal that CycR binds CyaC without the need of the nucleotides for the interaction.

**Fig. 4:**
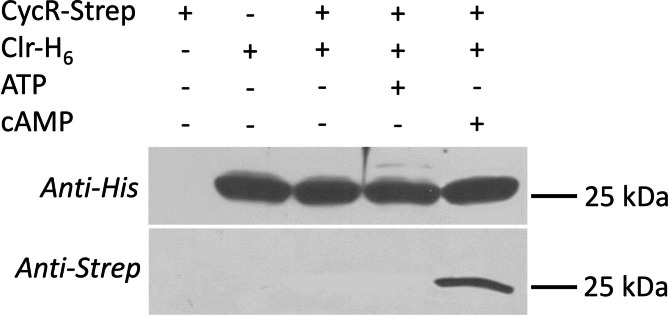
Co-sedimentation of CycR-Strep with Clr-His_6_ bound to magnetic particles. His_6_-Clr (29.3 µg) was incubated in 500 µl binding buffer containing CycR-Strep (28.3 µg), ATP (3.5 mM) and cAMP (0.5 mM) as indicated. After separation of the beads and washing, the proteins associated with the beads were separated by SDS-PAGE and tested by Western-blotting for Clr (anti-His_6_ antiserum, upper panel) and CycR (anti-Strep antiserum, lower panels) separately. Experiments were performed in two or more repeats. The positions of His_6_-Clr (29.3 kDa) and CycR-Strep (28.3 kDa) in the Westernblot are indicated by the protein marker of 25 kDa. The full length blots are shown in Figure S3

Furthermore, co-sedimentation of CycR with Clr-His_6_ attached to magnetic beads was analyzed (Fig. 4). Here, co-sedimentation of CycR with the Clr-His_6_-treated beads was observed, but only in the presence of cAMP, whereas presence of ATP had no positive effect. Overall, CyaC, CycR and Clr interact with each other, but the conditions for interaction differ: Interaction of CyaC with CycR is factor-independent, whereas interaction of Clr with CyaC, and CycR requires the assistance of ATP and the presence of cAMP, respectively.

### Binding kinetics of the interaction between CyaC and CycR

**Fig. 5:**
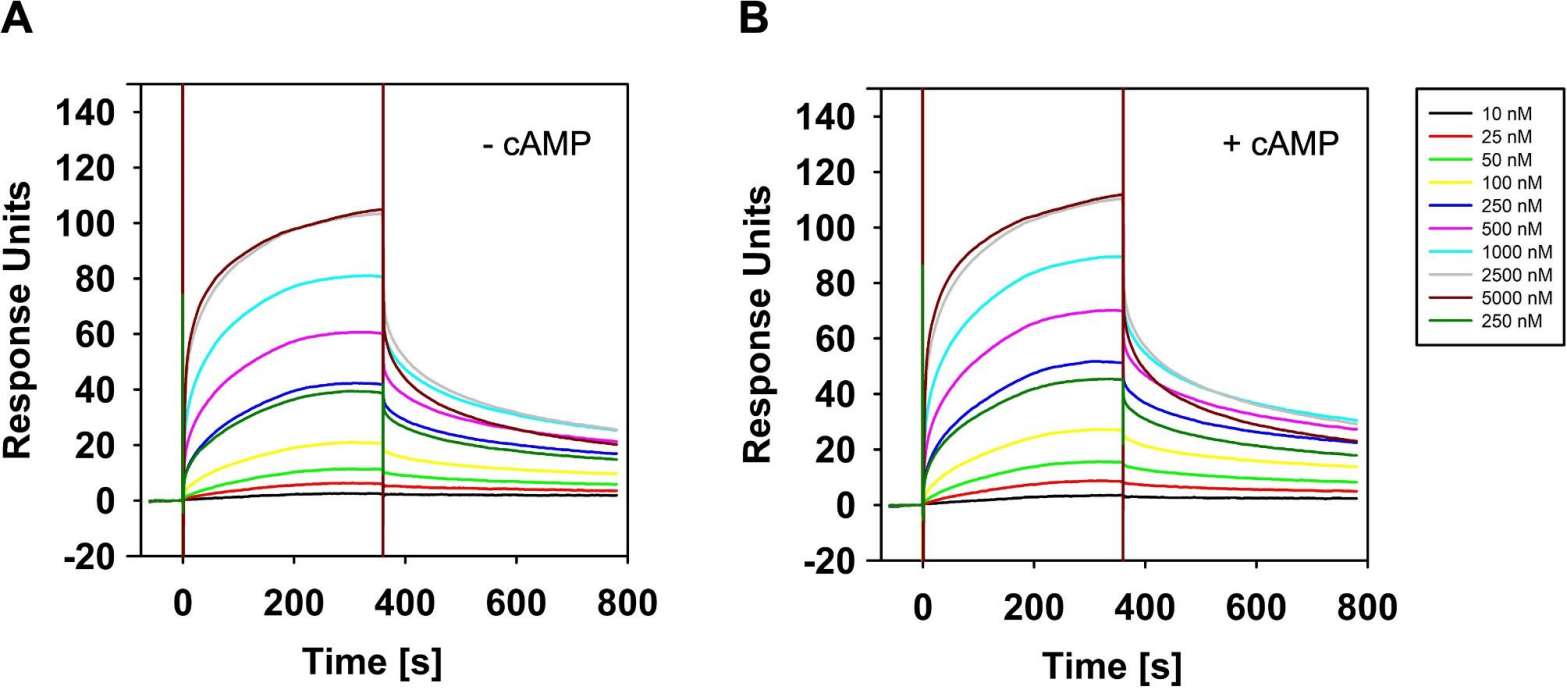
Binding of His_6_-CyaC to CycR-Strep. The graphs show SPR experiments, where CycR-Strep was captured onto a CM5 sensor chip previously immobilized with StrepTactin. First, approximately 200 Response Units of CycR-Strep were captured on the chip, and then different concentrations of His_6_-CyaC were injected [10 nM (dark green line), 25 nM (red line), 50 nM (light green line), 100 nM (yellow line), 2 × 250 nM (brown and blue line), 500 nM (turquoise line), 1.000 nM (light blue line), 2.500 nM (pink line), and 5.000 nM (brown line)]. Between each cycle, the chip was regenerated and new CycR-Strep was captured. The experiments were performed in the absence (**A**) and presence (**B**) of 500 µM cAMP. The pictures represent characteristic of at least three independent experiments

The preceding experiments indicate a CyaC×CycR×cAMP-Clr heterotrimeric complex, and that interaction of CyaC with CycR represents the backbone of the interaction, which is factor independent. Since BACTH is not quantitative respective to the interaction between two proteins as it represents an enzyme based reporter assay, slight changes in interaction strength or changes in binding kinetics cannot be monitored by this method. For that purpose, the binding kinetics between CyaC and CycR in absence and presence of cAMP was further analyzed by surface plasmon resonance (SPR) spectroscopy. As the first step, Strep-CycR was captured onto a sensor chip previously immobilized with StrepTactin. Then, different concentrations (10 nM-5,000 nM) of His_6_-CyaC were injected over the surface in the presence or absence of 500 µM cAMP, respectively. It could be observed that CyaC strongly interacted with CycR with an association rate of (*k*_*a*_) 2.9 × 10^4^ 1/s and a dissociation rate of (*k*_*d*_) = 4.6 × 10^− 3^ 1/M*s resulting in an overall affinity (K_D_) of 160 nM (Fig. 5A). As expected from the result of the co-sedimentation experiment of both proteins (Fig. 3), the SPR analysis showed that the presence of cAMP had no significant effect on the binding kinetics (*k*_*a*_ = 1.7 × 10^4^ 1/s; *k*_*d*_ = 1.9 × 10^− 3^ 1/M*s) and the affinity (K_D_=114 nM) (Fig. 5B). The R_max_ of approximately 100 Response Unites (RU) in absence or presence of cAMP revealed a 1:1 binding stoichiometry between ClrR (28 kDa) and CyaC (64 kDa) since approximately 200 RU of CyaC were captured in each cycle.

## Discussion

### The CyaC×CycR×cAMP-Clr signaling complex

CyaC interacts with the TetR-type regulator CycR and the CRP-like transcriptional regulator Clr when the proteins are produced in *E. coli*. The interaction is specified in vitro for the purified proteins. In vitro, interaction of CyaC with CycR is factor independent, but specific as shown by the association and dissociation constants of the SPR experiment. Second, interaction of CyaC with Clr requires ATP; it is lost or alleviated in the absence of ATP or in the active site mutant for ATP, CyaC^#^, as established in the co-sedimentation experiment. The experiment does not answer the question whether binding of ATP at CyaC, or the endogenously produced cAMP from CyaC, is required for the interaction. Interestingly, exogenous cAMP cannot replace the ATP (or the endogenous cAMP) in this experiment. Third, the interaction of CycR with Clr requires cAMP; the interaction of CycR with the cAMP-bound Clr is sufficiently stable for co-sedimentation. Therefore, it is assumed that the proteins form a highly specific CyaC×CycR×cAMP-Clr protein complex. The data for CyaC×Clr interaction indicate that in particular endogenous rather than exogenous cAMP is important for the complex formation. Interestingly, the co-sedimentation experiments suggest that CyaC×CycR form the more stable or preformed backbone of the complex, whereas the interaction of Clr with CyaC and with CycR is cofactor dependent.

CyaC prevails as dimer [5], which can be assumed also for the helix-turn-helix DNA-binding proteins [26] CycR and Clr. Moreover, the R_max_ determined by SPR revealed a 1:1 binding stoichiometry between CyaC and Clr, implying a complex of CyaC×CycR×cAMP-Clr with a 2:2:2 stoichiometry of the proteins.

**Fig. 6:**
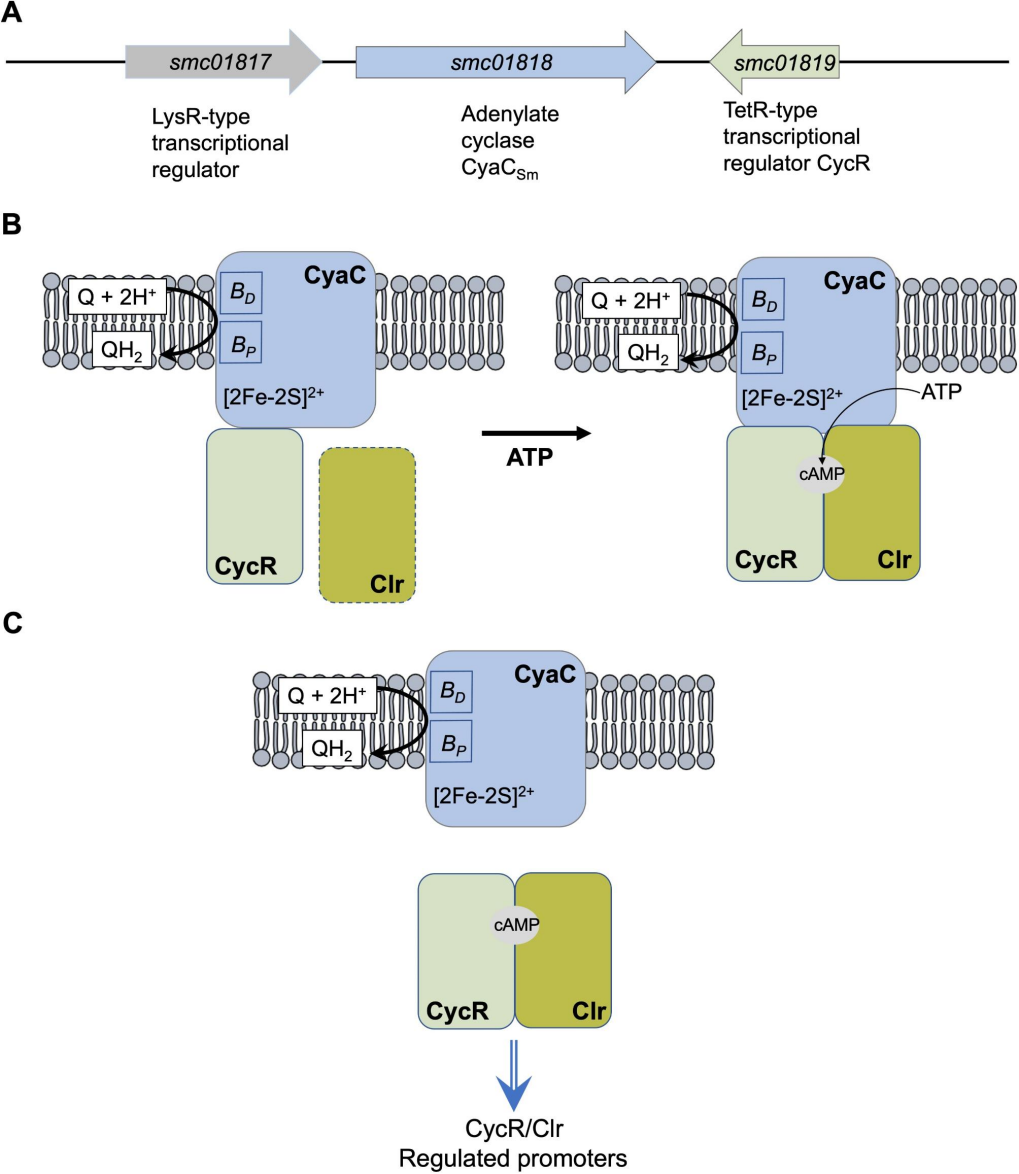
Scheme presenting gene arrangement at *cyaC***(A)**, the CyaC×CycR×cAMP-Clr sensor complex **(B)**, and the transcriptional regulatory complex CycR×cAMP-Clr **(C)**. **(A)** Adenylate cyclase CyaC forms with CycR the backbone of the CyaC×CycR×cAMP-Clr sensor complex. Cyclase activity of CyaC can be activated after oxidation by quinones (Q), resulting in cAMP formation (see [5]). Binding of Clr to CyaC and CycR is factor dependent. **(B)** The CycR×cAMP-Clr regulatory complex binds to CycR/Clr regulated promoters after detachment from CyaC and diffusion in the cytosol. The distal (B_D_) and proximal (B_P_) hemeB, and the [2Fe-2 S]^2+^ cluster of CyaC are indicated [5]

The K_D_ values for CyaC×CycR interaction prove that CycR is a specific part of the complex and suggests a joint role of the proteins in the regulation of a specific set of genes. Therefore, the previous putative protein TR01819 was renamed to CycR to indicate its role in CyaC-Clr dependent regulation. Indeed, we also tested protein pairs Clr/CycR and CyaC/Clr for interaction using SPR analysis. Unfortunately, in both cases no clear interaction was detectable using this method under the tested conditions. In SPR assays, one of the interactions partners has to be immobilized or captured via antibodies onto the chip surface. This can somehow affect the interaction between two proteins, presumably due to physical or sterical reasons, so that no interaction can be detected. Complex formation of CyaC with the transcription factors CycR and Clr enables specific signaling from the sensor CyaC to the regulators Clr and CycR. Here, the universal signaling molecule cAMP is used for the formation of a specific complex after activation of the cyclase activity of CyaC by the redox response [5]. Occlusion of the cAMP molecule within the complex is supposed to protect it from the other cAMP based signaling chains and to enable specific signaling. Thus, cAMP serves in this scheme as a trigger for the formation of a specific signaling complex (Fig. 6) rather than a diffusible secondary messenger. This mode allows specific signaling in a setting with multiple ACs and cAMP producing sites as known for *S. meliloti.* Overall, organization of the sensor CyaC together with two transcription factors in a complex provides a means to address a subset of genes from the large cAMP-Clr regulon. Furthermore, it provides a system for specific and selective cAMP signaling in a background of 28 ACs that use the same signaling molecule.

It is supposed that the CycR×cAMP-Clr protein complex is used for DNA binding and gene regulation after detachment from the (membrane associated) CyaC×CycR×cAMP-Clr complex (Fig. 6). Displacement of a gene regulator from a membrane-bound sensory complex for DNA binding has been observed directly for the response regulator DcuR that forms a sensory complex with sensor kinase DcuS [27]. For gene regulation, DcuR leaves the membrane-bound DcuR×DcuS complex and diffuses to the DNA in the cytosol. A similar scenario could apply to CyaC×CycR×cAMP-Clr.

### Regulation of a subset of the Clr regulon by CyaC×CycR×cAMP-Clr

Presence of cAMP-Clr and CycR in the complex suggests regulation of CyaC-responsive genes by two transcription factors. Regulation by two or more transcription factors is common for bacterial promoters, and often one of the regulators responds to an over-riding, and the second to a more limited stimulus. This type of co-regulation is characteristic for CRP-type regulators such as regulation of catabolite control by CRP of *E. coli* together with the lactose inducer LacI at the *lacZYA* operon [28], or the oxygen sensor FNR [29, 30] exerting co-dependent regulation of the *nir* operon together with the nitrate regulator NarL [31, 32]. CRP and LacI, FNR and NarL, respectively, cooperate at the promoters but are independent proteins, whereas cAMP-Clr and CycR are organized in a complex.

In the α-proteobacterium *S. meliloti*, which does not apply cAMP-CRP mediated catabolite control [33–35], cAMP and the CRP-like protein Clr play a major role in the regulation of *S. meliloti-Medicago* symbiosis [9, 10, 13]. More than 150 genes constitute the cAMP-Clr regulon and are positively or negatively regulated by cAMP-Clr [9, 13]. Within this large regulon, the ACs CyaD1, CyaD2 and CyaK cooperate with Clr and control expression of a set of genes [10]. As suggested here by interaction studies, CyaC is part of the cAMP-Clr regulon of *S. meliloti*. CyaC could be used to connect the cAMP-Clr regulon and the *S. meliloti-Medicago* symbiosis to the redox state of the respiratory quinones [5]. The redox status of respiratory quinones represents an important indicator for the energization and the redox status of the bacteria. These parameters have major implications on the life-style of bacteria, including symbiosis or the transition from free-living to endosymbiotic growth. Thus, the ubiquinone/ ubiquinol ratio is used for redox control of the ArcB-ArcA sensor system of *E. coli* [36, 37]. A comparable regulatory role of the redox state is feasible for *S. meliloti* symbiosis control via CyaC and CyaC×CycR×Clr, but other physiological roles cannot be ruled out.

## Conclusions

The organization of sensors like CyaC with transcription factors and a signaling molecule in a complex provides a means to form a linear and selective regulatory system within one organism, despite using the same secondary messenger like cAMP or c-di-GMP. Interestingly, *cyaC* of *S. meliloti* is preceded in addition to the downstream regulator gene *cycR*, by gene *smc01817* (Fig. 6A). The Smc01817 protein is a predicted LysR-type regulator, and the gene is supposedly co-transcribed [9] with *cyaC*. BACTH data suggest interaction of Smc01817 with CyaC (J. Wissig, R. Klein, G. Unden, unpublished). Therefore, complex formation between adenylate cyclases and transcriptional regulators could represent a common means for the formation of signaling complexes, and deserves further studies.

### Electronic supplementary material

Below is the link to the electronic supplementary material.

Supplementary Material 1

## Data Availability

All data generated or analyzed during this study are included in this published article or are available from the corresponding author on reasonable request.
