# Supplementary material for: A signaling complex of adenylate cyclase CyaC of Sinorhizobium meliloti with cAMP and the transcriptional regulators Clr and CycR
Source: BMC Microbiol. 2023 Aug 26;23:236. doi: 10.1186/s12866-023-02989-5 (PMC10463352; doi:10.1186/s12866-023-02989-5)
Supplement: Supplementary file 1 — Supplementary Material 1 [file 12866_2023_2989_MOESM1_ESM.docx]

**A signaling complex of adenylate cyclase CyaC of *Sinorhizobium meliloti* with cAMP and the transcriptional regulators Clr and CycR**

**SUPPLEMENTARY MATERIAL**

Robin Klein, Jannis Brehm, Juliane Wissig, Ralf Heermann*, and Gottfried Unden*

Institute of Molecular Physiology (imP), Microbiology and Biotechnology, Johannes Gutenberg University, Biocenter II, Hanns-Dieter-Hüsch-Weg 17, 55128 Mainz, Germany

*Corresponding authors:

[heermann@uni-mainz.de](mailto:heermann@uni-mainz.de)

[unden@uni-mainz.de](mailto:unden@uni-mainz.de)

Keywords: second messenger, CyaC, cyclic AMP, TetR-type regulator, cyclic-AMP-receptor protein CRP, *Sinorhizobium meliloti*, *Ensifer meliloti*


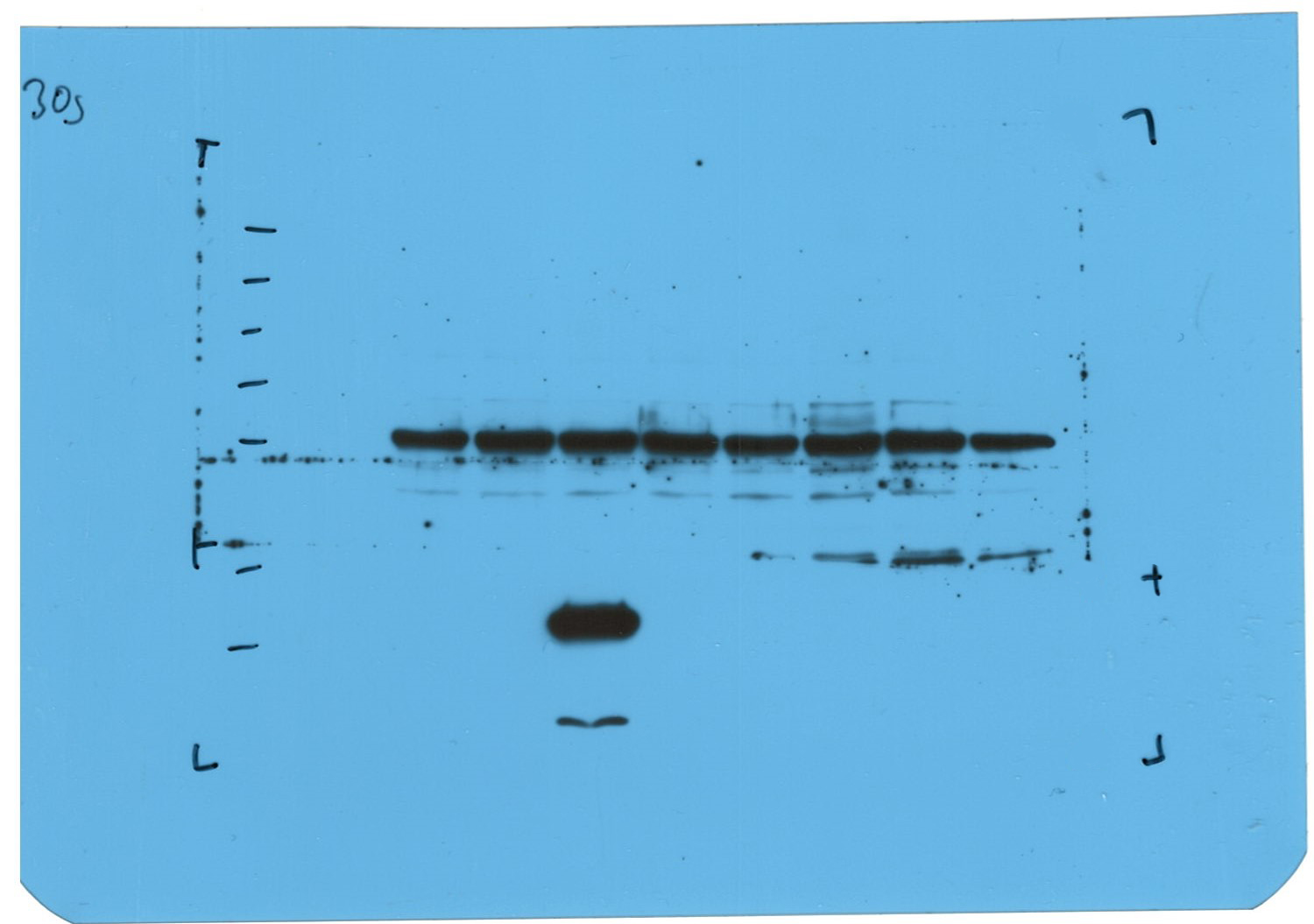


**Figure S1. Co-sedimentation of Clr-Strep with His_6_-CyaC and His_6_-CyaC^#^ bound to magnetic beads.** Original blots that are depicted in Figure 2A (lanes 1-5) and 2B (lanes 6-10). Upper panel: anti-His, lower panel: anti-Strep.


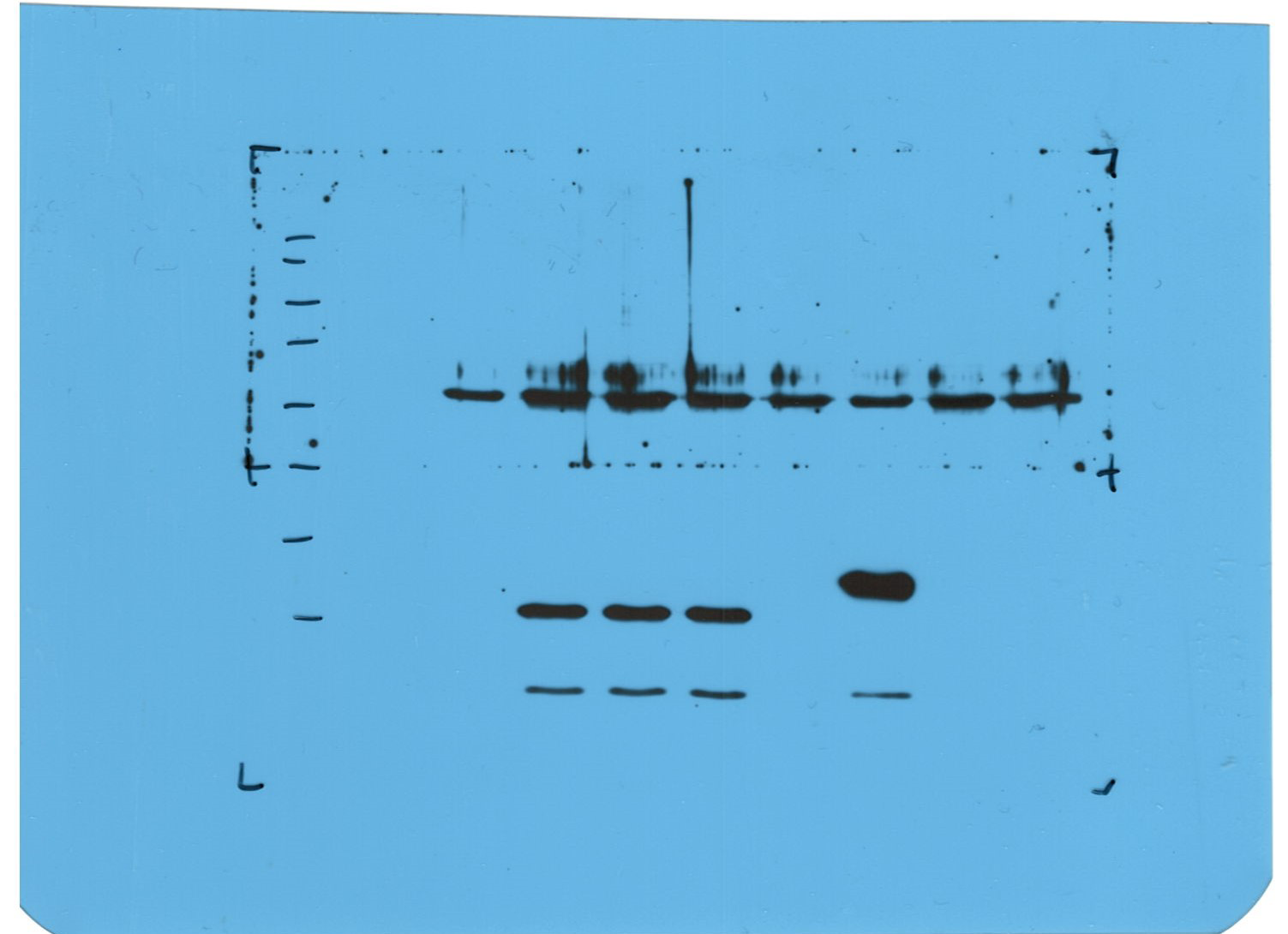


**Figure S2. Co-sedimentation of CycR-Strep with His_6_-CyaC bound to magnetic particles.** Original blots that are depicted in Figure 3 (lanes 1-5). Upper panel: anti-His, lower panel: anti-Strep.


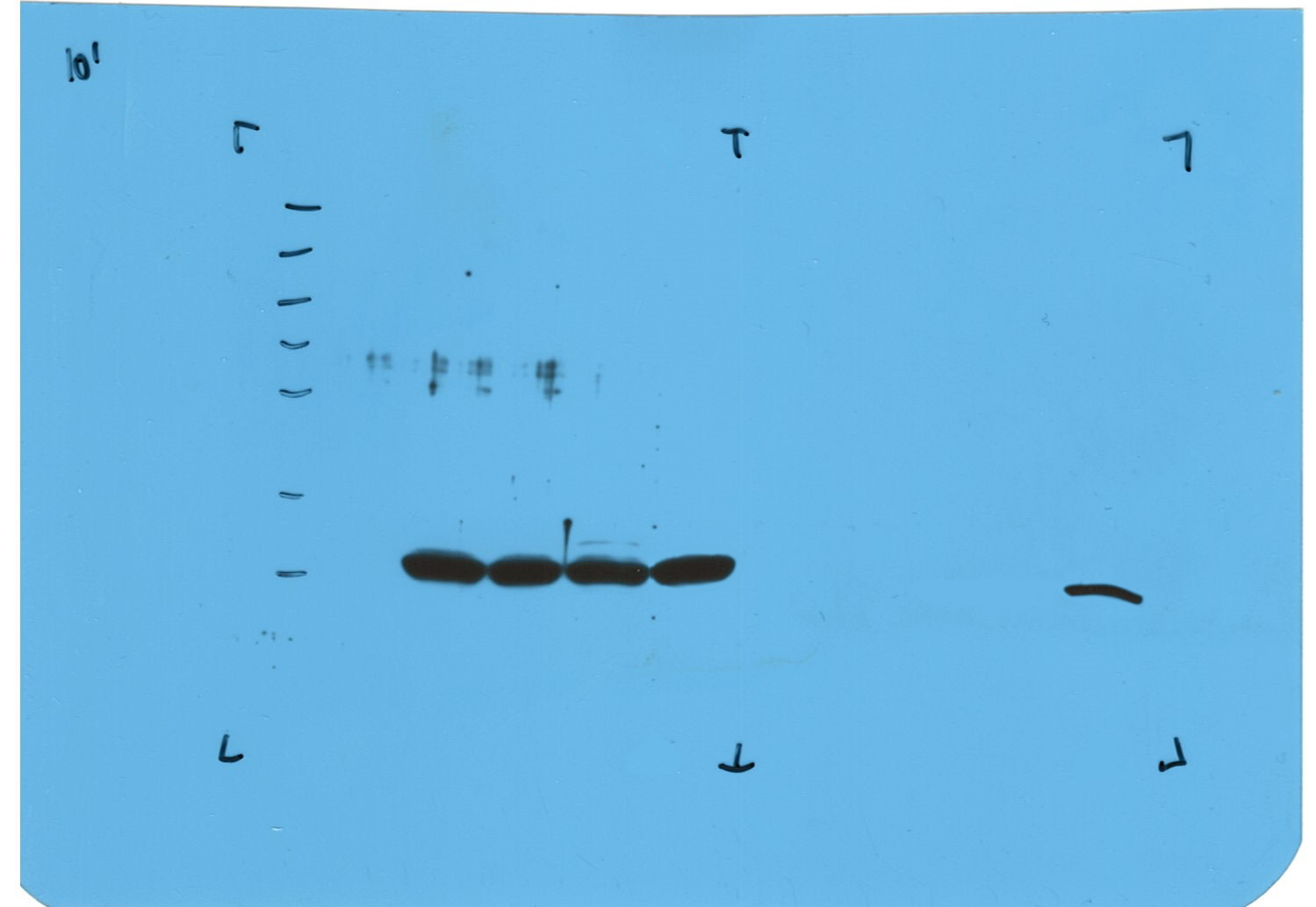


**Figure S3. Co-sedimentation of CycR-Strep with Clr-His_6_ bound to magnetic particles.** Original blots that are depicted in Figure 4. Lanes 1-5: anti-His, lanes 6-10: anti-Strep.
